# Regional Anesthetic and Analgesic Techniques for Clavicle Fractures and Clavicle Surgeries: Part 1—A Scoping Review

**DOI:** 10.3390/healthcare10081487

**Published:** 2022-08-07

**Authors:** Chang Chuan Melvin Lee, Zhi Yuen Beh, Chong Boon Lua, Kailing Peng, Shahridan Mohd Fathil, Jin-De Hou, Jui-An Lin

**Affiliations:** 1 Department of Anesthesia, Toowoomba Base Hospital, Darling Downs Hospital and Health Service, Brisbane, QLD 4350, Australia; 2 Rural Clinical School, Toowoomba Regional Clinical Unit, University of Queensland, Brisbane, QLD 4350, Australia; 3 Department of Anesthesia, National University Health System, Singapore 119074, Singapore; 4 Center for Regional Anesthesia and Pain Medicine, Chung Shan Medical University Hospital, Taichung 40201, Taiwan; 5 OSC Orthopaedic Specialist Centre, Subang Jaya 47600, Malaysia; 6 Department of Anesthesiology, Assunta Hospital, Petaling Jaya 46990, Selangor, Malaysia; 7 Department of Anesthesiology, Gleneagles Hospital Medini, Nusajaya 79250, Malaysia; 8 Center for Regional Anesthesia and Pain Medicine, Wan Fang Hospital, Taipei Medical University, Taipei 116, Taiwan; 9 Division of Anesthesiology, Hualien Armed Forces General Hospital, Hualien 97144, Taiwan; 10 Department of Anesthesiology, School of Medicine, National Defense Medical Center, Taipei 11490, Taiwan; 11 Department of Anesthesiology, Wan Fang Hospital, Taipei Medical University, Taipei 116, Taiwan; 12 Department of Anesthesiology, School of Medicine, College of Medicine, Taipei Medical University, Taipei 110, Taiwan; 13 Pain Research Center, Wan Fang Hospital, Taipei Medical University, Taipei 116, Taiwan; 14 Department of Anesthesiology, School of Medicine, Chung Shan Medical University, Taichung 40201, Taiwan; 15 Department of Anesthesiology, Chung Shan Medical University Hospital, Taichung 40201, Taiwan

**Keywords:** analgesia, anesthesia, clavicle, fractures, bone, motor activity:motor-sparing, nerve block, pain, postoperative

## Abstract

**Objective.** Clavicle fractures are common injuries potentially associated with significant perioperative pain. However, this region’s complex sensory innervation poses a challenge for regional anesthetic or analgesic (RA) techniques. We conducted this scoping review to summarize the current literature, particularly with regards to motor-sparing techniques. **Methods.** A scoping review was carried out in accordance with the Joanna Briggs Institute’s framework. All articles describing the use of RA for clavicle fractures or surgery were included. PubMed^®^, Ovid MEDLINE^®^, EMBASE^®^, Scopus^®^, CINAHL^®^, and the Cochrane database were searched without language restrictions. **Results.** Database searches identified 845 articles, 44 of which were included in this review, with a combined patient total of 3161. We included all peer-reviewed publications containing clinical data and summarized the findings. **Conclusions.** Current evidence of RA techniques in clavicle surgery is heterogeneous, with different approaches used to overcome the overlapping sensory innervation. The literature largely comprises case reports/series, with several randomized controlled trials. Intermediate cervical plexus block is the regional technique of choice for clavicle surgery, and can provide reliable surgical anesthesia when combined with an interscalene block. Cervical plexus block can provide motor-sparing analgesia following clavicle surgery. Promising alternatives include the clavipectoral block, which is a novel motor-sparing regional technique. Further studies are required to determine the efficacy and safety of various techniques.

## 1. Background

Clavicle fractures are the most common injury involving the shoulder girdle, accounting for approximately 2.6% of all fractures and 44% of those in the shoulder girdle [1,2]. The most affected demographic are young males, and the midshaft is the most common fracture site (69–82%) [1,2]. Although stable fractures may be conservatively managed, unstable or displaced fractures, as well as those associated with pain, functional limitation, and/or neurovascular compromise, may require further treatment including fracture fixation [2,3,4,5].

Fractures of the clavicle are associated with significant pre- and post-operative pain [6]. Although general anesthesia (GA) is commonly employed, clavicle fixation surgery performed under regional anesthesia (RA) with or without sedation is feasible and has been previously reported [7,8]. Multimodal analgesia utilizing surgical site infiltration, and different permutations of peripheral nerve blocks targeting the cervical plexus, brachial plexus, or their branches, have been described in the literature [6,9,10]. Given the breadth of techniques reported in the literature, stronger evidence to determine the most appropriate regional technique for emergency department and perioperative (surgical fixation) settings is required [11]. Furthermore, with the widespread availability of ultrasound platforms and the adoption of real-time ultrasound guidance as the *de facto* gold standard, sonography has contributed to the safety of RA, improved the efficacy of existing techniques, and paved the emergence of novel fascial plane blocks, such as the clavipectoral fascial plane (CPB) block and pectoral nerve block (PECS) [12,13,14,15,16]. Presently, many centers prefer GA with or without RA due to the region’s complex sensory innervation, and limited experience with RA as the main technique in this patient population.

Nonetheless, despite the attractiveness of RA, the preferred RA technique at present involves a superficial cervical plexus block (SCPB) and interscalene brachial plexus block (ISB) if complete anesthetic or analgesic cover is required [13]. Due to its close anatomical proximity, the latter is associated with motor blockade of the phrenic nerve—leading to hemidiaphragmatic paresis and the potential for respiratory compromise in susceptible patients. Furthermore, blockade of brachial plexus outflow leads to an insensate, immobile limb that can potentially compromise function and delay recovery, requires protection from injury, and can affect surgical assessment of neurovascular function [17].

In this scoping review, we sought to examine the current evidence of various regional techniques for anesthesia and analgesia in clavicle fracture surgery, with a particular emphasis on the latest approaches utilizing ultrasound guidance which may allow preferential sensory blockade or analgesia whilst preserving motor function of the upper limb.

## 2. Materials and Methods

We conducted a scoping review in accordance with the preferred reporting items for systematic reviews and meta-analysis (PRISMA) framework recommended by the Joanna Briggs Institute [18]. Unlike systematic reviews and meta-analyses, a registered protocol is not required for scoping reviews. A scoping review was chosen instead of a systematic review as the evidence relating to regional anesthetic and analgesic techniques in clavicular fracture surgery have not been comprehensively reviewed, and the diverse range of study designs makes quantitative synthesis difficult.

**Search strategy.** A comprehensive literature search using six electronic database search engines was performed. PubMed^®^, Ovid MEDLINE^®^, Excerpta Medica dataBASE (EMBASE^®^), Scopus^®^, Cumulative Index to Nursing and Allied Health Literature (CINAHL^®^), and the Cochrane database of systematic reviews were searched for articles published from 2001 to 2022. We chose this time frame to include relatively up-to-date articles, and there was a paucity of literature prior to this date; yet, it still captured landmark and nerve stimulator-guided blocks, as well as the paradigm-shift towards ultrasound-guided techniques. The terms ‘clavicle’, ‘fracture’, ‘regional anesthesia’, ‘pain’, and ‘analgesia’, as well as their synonyms, and combinations of these keywords, were used to develop the search string, the concepts of which are provided in Supplementary Table S1. The search terms were joined by Boolean operators. The search was performed from February to March 2022. In addition, a manual search of citations and references was performed to further identify relevant articles. No limits were placed on our search.

**Eligibility criteria**. Articles eligible for inclusion were those describing any regional anesthetic or analgesic technique employed for clavicular pain in the context of a fracture and/or surgery in adults or children. We defined the clavicular region from the clavicle’s articulation between the sternum medially and the acromion laterally. We did not place any restrictions on the article type as long as it provided clinical data. There were also no restrictions placed on the number of patients or language. 

**Study selection and reliability.** Articles were selected for inclusion in the review if they fulfilled the above eligibility criteria and contained clinical data. Our scoping review was aimed to broadly capture the available evidence. Therefore, we included original re- search, correspondences, case reports, case series, conference abstracts, and presentations, as these could potentially contain sufficient clinical detail to evaluate the current evidence for regional anesthesia in clavicular fracture surgery. 

All titles and abstracts were screened independently by two authors against a set of *a priori* defined eligibility criteria. Potentially relevant studies were selected for full-text analysis. The reference lists of the included studies were also examined for relevant articles in order to ensure the literature saturation. A summary of the inclusion and exclusion criteria is presented in Table 1. Studies that had not reported clinical data on regional anesthesia in clavicular fracture fixation were excluded. Disagreements were resolved by consensus or consultation from a third senior reviewer.

**Data charting.** A *pro forma* was created for data charting with the following fields relevant to the present review, many of which are summarized and presented in Table 2: (1) authors and year of publication, (2) country, (3) article type, (4) study design and participant size, (5) regional anesthetic technique or combination of techniques, (6) control or comparator technique, (7) volumes and type of LA, (8) the use of any sedation or general anesthesia as part of the planned study design, (9) main outcomes, and (10) reported complications. This *pro forma* was continuously updated throughout the data extraction process due to the heterogeneity of study designs, interventions, and reported outcomes. Two authors independently charted the data from each included article. Disagreements were resolved by consensus or consultation from a third senior reviewer. 

## 3. Results

**Figure 1:**
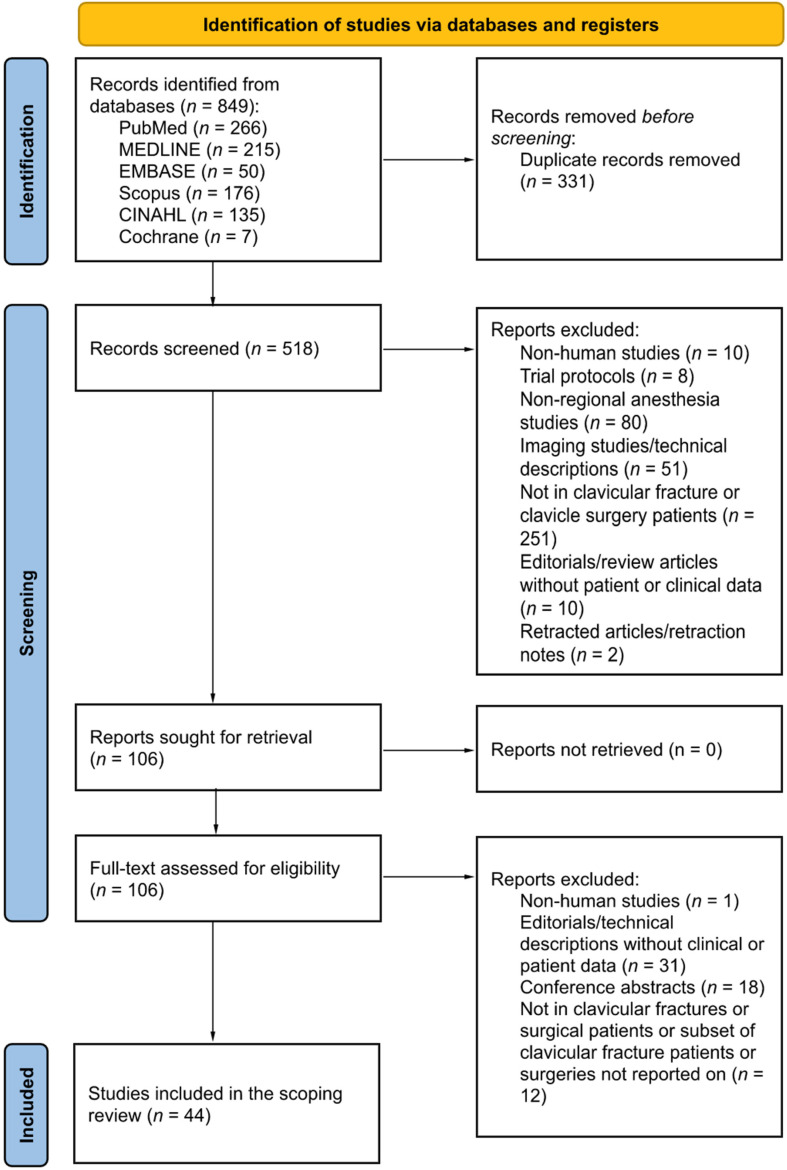
A PRISMA (preferred reporting items for systematic reviews and meta-analyses) flow diagram for studies included and excluded from the present review.

**Characteristics of included studies.** The search returned a total of 849 articles. After removal of duplicates, 518 unique citations were identified for screening. Following a thorough review of the titles, abstracts, full-text, and reference lists, we included and extracted data from a total of 44 studies and/or reports containing patient data, spanning a total of 3161 patients with clavicular fractures who received some form of regional anesthesia and analgesia. We excluded one retracted article and its retraction note from the final list of 44 studies [19,20,21,22,23,24,25,26,27,28,29,30,31,32,33,34,35,36,37,38,39,40,41,42,43,44,45,46,47,48,49,50,51,52]. There were 5 RCTs, 5 prospective observational studies, 5 retrospective observational studies, and 29 case reports/case series included in this scoping review (Figure 1 and Table 2).

**Table 2:** A summary of randomized controlled and observational studies included in this scoping review [6,7,8,13,21,23,24,25,35,36,37,43,44,45,48,53].

Reference (Year), Country	Study Design	Sample Size	Fracture Location	Type of Block	Needle Guidance	LA Type and Volume	Anesthetic Technique	Perioperative Analgesia Regime	Outcome(s)
[6] Yoo and colleagues (2018), South Korea	Retrospective observational	50 (25 + 25)	Midshaft and distal	GA and surgical site infiltration vs. GA alone	LM	30 mL of injectate comprising of 300 mg ropivacaine, 5 mg morphine sulphate, 1 mg adrenaline, and 20 mL 0.9% sodium chloride (total volume 61.5 mL)	GA	IV Fentanyl and ketorolac PCA for 24 h, paracetamol, tramadol, pregabalin	Significant ↓ pain scores and Tramadol requirement up to 24 h with surgical site infiltration. Infiltration ↓ DHEA-S levels at 72 h (*p* = 0.046), but no differences in insulin or fibrinogen.
[7] Reverdy (2015), France	Prospective observational	12	NR	SCPB and ISB	US	1% mepivacaine or 0.75% ropivacaine, median volume 20 mL (range 16 to 40 mL)	Sedation or awake	Paracetamol, ketoprofen	Sedation required in 2 cases. Mean VAS 2 (range 0 to 3) on day 0 and 1.
[8] Banerjee and colleagues (2019), India	RCT	60 (30 + 30)	NR	SCPB and ISB vs. GA alone	US	SCPB: 5 mL 2% lignocaine with adrenaline and 5 mL 0.5% bupivacaine ISB: 8 mL 2% lignocaine with adrenaline and 8 mL 0.5% bupivacaine	Awake vs. GA	Fentanyl, paracetamol, tramadol	SCPB + ISB ↓ post-operative pain scores, time spent in recovery, and postoperative opioid requirement vs. GA alone. SCPB + ISB ↑ interval to first occurrence of pain (324.7 min) vs. GA.
[13] Zhuo and colleagues (2022), China	RCT	40	Midshaft	ICPB and ISB vs. CPB and ISB	US	ICPB: 5 mL 0.375% ropivacaine ISB: 20 mL 0.375% ropivacaine CPB: 20 mL 0.375% ropivacaine	Awake	NR	No difference in pain scores in recovery. No block failures. Residual motor block in 8 of 20 (40.0%) patients from the ICPB + ISB group at 4 h post-block. ICPB + ISB ↓ in FVC, FEV1, PEFR, and hemi-diaphragmatic excursion in compared to ICPB + CPB.
[21] Abdelghany and colleagues (2021), Egypt	RCT	70 (35 + 35)	NR	SCPB vs. SCPB and ISB	US	SCPB: 10 mL 0.25% bupivacaine ISB: 15 mL 0.25% bupivacaine	GA	Fentanyl *, paracetamol, morphine	SCPB alone significantly ↓ the incidence of phrenic nerve palsy (22.9% vs. 2.9%, *p* value 0.03). No difference in intraoperative or post-operative pain scores, or analgesic/anaesthetic requirement. No difference in the incidence of perioperative complications or patient satisfaction. Horner’s syndrome (5.7% vs. 2.9%).
[23] Arjun and colleagues (2020), India	Randomised, double-blind RCT	50 (25 + 25)	26 Midshaft 24 Distal	SCPB and ISB vs. ICPB and ISB	US	SCPB or ICPB: 10 mL 0.5% bupivacaine ISB: 10 mL 0.5% bupivacaine	Sedation	Tramadol	100% block success with ICP and ISB vs. 5 patients (20%) with block failure in SCPB + ISB group. Faster onset and ↑ duration of analgesia (540 vs. 342 min) with ICPB + ISB vs. SCPB + ISB.
[24] Olofsson and colleagues (2020), Switzerland	Prospective case-control	126 (50 + 76)	95 Midshaft 31 Distal	ISB with GA vs. GA alone	US	ISB: 20 mL 0.5% bupivacaine	GA	Sufentanil *, morphine, paracetamol, oxycodone	Patients with ISB had ↓ pain scores in recovery (mean difference 1.7 points) and significantly ↓ analgesic requirement (mean difference 8.3 mg). Patients with ISB also had ↓ intraoperative Sufentanil requirements. ISB ↓ post-operative nausea and vomiting (4% vs. 17%) vs. GA alone (not statistically significant)
[25] Ryan and colleagues (2020), USA	Retrospective observational	110 (52 + 58)	90 Midshaft 20 Distal	SCPB and ISB vs. ISB with GA	LM	SCPB: 10 mL 0.5% bupivacaine ISB: 20 mL 0.5% bupivacaine with adrenaline	Awake vs. GA	No standardized analgesia regimen	No conversion from block group to GA. SCPB + ISB ↓ intraoperative Fentanyl (141 µg vs. 207 µg, *p* = 0.002) vs. ISB + GA
[35] Beletsky and colleagues (2020), USA	Retrospective observational	2300 (346 + 1954)	NR	NR	NR	NR	NR	NR	Regional anesthesia use is associated with ↑ odds (1.70, *p* < 0.01) for same-day discharge.
[36] Neha Gupta and colleagues (2019), India	RCT	60 (30 + 30)	NR	ISB alone vs. ISB and SCPB	NR	SCPB: 0.5 mg.kg^−1^ bupivacaine with 1 mg·kg^−1^ lignocaine to ≥10 mL ISB: 1 mg·kg^−1^ bupivacaine with 3 mg·kg^−1^ lignocaine to ≥20 mL	Sedation	Fentanyl *	ISB-only group required ↑ supplementary LA at the medial end (16.7% vs. none) and had ↑ conversion to GA (10.0% vs. 3.3%) Hoarseness of voice: 36.7% of the entire cohort.
[37] Kaciroglu and colleagues (2019), Turkey	Retrospective	16	1 Medial 3 Midshaft 6 Lateral	SCPB and ISB	US	SCPB: 5 mL 2% lignocaine and 5 mL 0.5% bupivacaine ISB: 7.5 mL 2% lignocaine and 7.5 mL 0.5% bupivacaine	Mixed	NR	All patients with lateral fractures underwent surgery awake. 1 patient required GA, and 4 patients required sedation. Mean duration of motor block and analgesia 213 and 259 min respectively.
[43] Rajbanshi and colleagues (2018), Nepal	Randomised prospective comparative study	60 (30 + 30)	NR	SCPB and ISB vs. SCPB and SpC	US	SCPB: 10 mL 0.25% bupivacaine ISB: 20 mL 0.25% bupivacaine SpC: 20 mL 0.25% bupivacaine	Sedation	Fentanyl, paracetamol	2 cases from the SCPB + ISB group and 3 cases from the SCPB + SpC group required conversion to GA. No difference in duration of motor block: SCPB + ISB group vs. SCPB + SpC group (347 min vs. 392 min, *p* = 0.16) No difference in Horner’s syndrome (7.3% vs. 1.8%) or hoarseness of voice (7.3% vs. 3.6%).
[44] Ho and colleagues (2018), Canada	Prospective observational	7	NR	SCPB	US	8–14 mL 0.25–0.5% bupivacaine with adrenaline	Not applicable	NR	Emergency department study for analgesia provision, with a subset of 7 patients with clavicle fractures. Mean ↓ in pain scores by 6.29 points or 73.5%. Out of the entire cohort of 27 patients, 1 patient developed hemidiaphragmatic paresis, and another developed hoarseness.
[45] Balaban and colleagues (2018), Turkey	Retrospective observational	12	NR	SCPB and ISB	US	SCPB: 0.25 mL·kg^−1^ 0.5% bupivacaine ISB: 0.25 mL·kg^−1^ 0.5% bupivacaine	Sedation	Tramadol	One patient felt mild pain at the start of surgery, and another patient required deeper sedation during manipulation of the clavicle.
[48] Contractor and colleagues (2016), India	Prospective	30	NR	SCPB and ISB	Unclear, possibly US	SCPB: 10 mL 0.25% bupivacaine ISB: 10–15 mL 1.5% lignocaine with adrenaline plus 5–10 mL 0.5% bupivacaine	Sedation	NR	Mean duration of analgesia 277 min. Incidence of Horner’s syndrome (26.7%), and hoarseness of voice (16.7%).
[53] Kuchyn (2013), Russia	Randomised controlled trial	60	NR	SCPB and ISB (nerve stimulator vs. ultrasound guided)	LM	SCPB: 0.5% lignocaine ISB: 1% lignocaine, 0.25% bupivacaine Total LA volume 30–40 mL	Sedation	NR	Ultrasound guidance ↓ conversion to GA vs. nerve stimulation (*p* = 0.024) (failure ↑ with nerve stimulation alone, odds ratio 13.16)

Abbreviations are as follows: ICPB, intermediate cervical plexus block; ISB, interscalene brachial plexus block; CPB, clavipectoral plane block; RCT, randomized controlled trial; LA, local anesthetic; IV, intravenous; PCA, patient controlled analgesia; FVC, forced vital capacity; FEV_1_, forced expiratory volume in 1 s; PEFR, peak expiratory flow rate; NR, not reported; SCPB, superficial cervical plexus block; SpC, supraclavicular brachial plexus block; WALANT, wide-awake local anesthesia no tourniquet; DCP, deep cervical plexus; SpN, supraclavicular nerve; GA, general anesthesia; NB, nerve block; ACJ, acromioclavicular joint; PECS, pectoralis nerve; VAS, visual analogue scale; NRS, numerical rating scale; POD, postoperative day; DHEA-S, dehydroepiandrosterone sulfate.

**Cervical plexus block nomenclature.** Although collectively described as a SCPB, by definition, a SCPB essentially involves a subcutaneous infiltration of LA along the posterior border of the sternocleidomastoid (SCM); once the needle pierces the investing fascia of the neck (while remaining superficial to the deep cervical fascia), it is termed an intermediate cervical plexus block (ICPB) [54,55,56,57,58]. We found significant variability in the in-text description of a SCPB amongst the included literature—a fundamental problem when trying to elucidate the efficacy of a SCPB in this context if one were to consider the investing fascia a potential barrier to deeper spread of LA compared to a subcutaneous injection. Thus, we attempted to classify the studies, based on a unifying nomenclature system, into superficial and intermediate cervical plexus blocks based on the actual in-text description or included sonographic image based on the site of LA deposition relative to the investing fascia of the neck and prevertebral fascia (Table 3). Most studies purportedly, in-text, included a SCPB (28 articles). However, considering the nomenclature differences above, we contend that at least 14 of these studies described an ICPB, while 5 studies performed a SCPB (Table 3) [7,8,13,15,21,23,25,28,32,33,34,36,37,38,39,42,43,45,46,47,48,49,50,51,52,53,59,60]. Taking the in-text description at face value, two articles seemed to describe a deep cervical plexus block with deposition of LA below the prevertebral fascia, while the remaining eight studies did not provide sufficient detail to classify the block [8,15,28,33,34,36,37,48,60]. In the remainder of this manuscript, we have referred to cervical plexus blocks using this revised nomenclature as far as possible for consistency. Where insufficient detail was provided for classification, we have referred to the technique broadly as a “cervical plexus block”.

**Regional anesthetic approaches.** The studies and reports included in our present review are presented in Table 2 and Table S2.

A number of articles (27) have reported clavicle fixation surgery in awake or sedated patients (Table 2 and Table S2), and most reports or studies which employed RA in awake or sedated patients utilized a combination of two techniques.

A total of 29 studies investigated the use of a cervical plexus block (Table 2 and Table 3 and Table S2). Cervical plexus blockade was also commonly used in combination with another nerve or fascial plane block. The most common combined technique utilized cervical plexus blockade with an ISB, as reported in 18 studies [7,8,13,21,23,25,32,36,37,38,39,43,45,46,48,51,52,53]. Of these, five studies utilized a SCPB, and nine studies described an ICPB [7,13,21,23,25,32,38,39,45,46,48,51,52,53]. A single case report described a deep cervical plexus block [60]; however, based on our standardized nomenclature, two other studies had in-text descriptions suggestive of a deep cervical plexus block [8,51].

The interscalene approach to the brachial plexus was the next most common technique, as described in 21 studies [7,8,13,21,23,24,25,34,36,37,38,39,40,41,43,45,46,48,51,52,53]. The supraclavicular approach to the brachial plexus was described only in a case report and a prospective randomized study, and in both cases this was combined with an ICPB [32,43].

A small number of studies investigated the use of specific nerve blocks, or more targeted RA approaches. A selective supraclavicular nerve block was described in four studies [10,26,40,41], and a targeted C5 and/or C6 nerve root blocks in another four studies [26,47,50,59]. We found three case reports describing the use of a superior trunk block, including one in which superior trunk blockade was the sole anesthetic technique [9,10,42]; in the other two reports this was combined with a supraclavicular nerve block or ICPB [9,10,42].

Several studies have examined the use of fascial plane blocks. The use of a CPB was described in seven studies, which was combined with a cervical plexus block in three studies [13,15,28]. Its use as a sole technique was described in two case reports [14,20], and a combination of ICPB and CPB used as the sole technique in an RCT [13]. One study described the pectoralis block (PECS) I in one study, while another combined a cervical plexus block with a PECS II block, both in patients under GA [16,33].

Lastly, awake clavicle fracture fixation surgery under tumescent local anesthesia (LA) alone has also been described in a case report, as well as in a case series [22,27].

## 4. Discussion

We conducted this present scoping review in order to comprehensively review the trends in RA techniques employed for clavicle fractures and clavicle surgery, and elucidate motor-sparing techniques which may provide reliable anesthesia and/or analgesia. In this section, we discuss the issues and challenges surrounding RA in this patient population, as well as summarize our findings.

**Heterogeneity of studies**. We found that the literature comprises of heterogeneous studies, which likely stems from different approaches used to overcome the overlapping sensory innervation in this region. There is a paucity of large randomized studies, and the literature largely comprises case reports and case series, with several retrospective studies or small randomized trials. This is unsurprising considering the complex, overlapping, and variable sensory innervation of the clavicular region that remains disputed [61]. This is further compounded by varying fracture locations, complexity, and approach over areas of mixed sensory innervation. Patients with clavicle fractures may also have other injuries which may place them at increased risk of complications from specific RA techniques. Lastly, inter-individual and intra-individual variability in sensory innervation and pain perception add another layer of complexity to the provision of anesthetic care [62]. At present, the heterogeneity of published studies makes any form of quantitative synthesis difficult to perform.

**Applied anatomy and complexity of innervation.** The superficial cervical plexus originates from the ventral rami of C1 to C4, which give rise to four terminal branches. Amongst these, the supraclavicular nerve is the most relevant to the innervation of the clavicular region, and provides sensory innervation to the skin overlying the clavicle [63]. The innervation of the clavicle itself is a subject of much more debate. The periosteum receives a rich sensory innervation via fibers from motor, articular, and cutaneous nerve branches, as well as from nerves following nutrient arteries [11,64]. The supraclavicular nerve supplies the entire length of the clavicle periosteum on its cephalad and ventral surfaces, while the dorsal and caudal surfaces demonstrate overlapping innervation by the subclavian nerve (also known as the nerve to the subclavius) on the middle and medial thirds, and by the lateral pectoral nerve on the caudal aspect of the middle and lateral thirds [11,63,64]. The sternoclavicular joint is supplied by the supraclavicular nerve, and the acromioclavicular joint by both the acromioclavicular branch of the supraclavicular nerve, and the lateral pectoral nerve [62,63,64,65]. It is also postulated that the medial part of the clavicle receives sensory innervation by the spinal accessory nerve, and the lateral aspect from the subscapular and axillary nerves [64]. Based on this complex innervation pattern, it is difficult for any single block to completely anesthetize the entirety of the clavicular region.

**Surgical technique and approaches.** The permutation of involved segments and types of surgical procedures across areas of overlapping sensory innervation warrant consideration when choosing an appropriate technique. The middle third of the clavicle is the most commonly involved [1,2,66]. The open reduction and surgical fixation of clavicle fractures are commonly performed, and the patient is usually positioned in a supine or modified beach-chair position. Indications for surgical fixation include open fractures, neurovascular compromise, severe angulation or displacement with a risk of cutaneous perforation, or symptomatic non-union. [67,68,69,70,71]. Either a longitudinal incision along the subcutaneous border of the clavicle or a vertical (necklace) incision along the Langer lines may be used for osteosynthesis using pre-contoured plates, although smaller skin incisions may be used in a minimally invasive approach with plate osteosynthesis or intramedullary fixation devices [72,73,74,75]. Unstable distal fractures may further require coracoclavicular repair, tension-band wiring, reconstruction, hook plating, or transacromial pinning [75]. Furthermore, patients might have other concomitant injuries which can increase the risk of specific techniques, such as respiratory compromise or a contralateral chest injury or pneumothorax.

**Cervical plexus block.** Although multiple studies have investigated cervical plexus blockade in combination with ISB, comparative studies investigating cervical plexus blockade with GA versus GA alone are limited [7,21,24,25,32,38,39,45,46,48,51]. However, an RCT by Abdelghany et al. suggests that for clavicle surgery performed under GA, a cervical plexus blockade might be sufficient for analgesia without the need for ISB (Table 3) (Supplementary Table S2) [21]. A cervical plexus block alone is associated with a lower incidence of phrenic nerve palsy (22.9% vs. 2.9%), and hoarseness, whilst providing comparable postoperative pain control with those who received combined ICPB and ISB plus GA [21,36].

The next question is whether an efficacy difference exists between the SCPB and ICPB. Interestingly, a single RCT (50 cases) comparing SCPB and ISB versus an ICPB with an ISB by Arjun et al. reported a 20% block failure rate in the group receiving a SCPB and ISB, despite the use of ultrasound [23]. Similar studies were not elucidated during our literature search for comparison.

A case report by Choi et al. (Table 3 and Table S2) described a deep cervical plexus block, which was combined with a SCPB for postoperative analgesia, in which the patient remained pain free for 14 h with no reported complications [55]. No larger series or comparative studies were found.

**Interscalene brachial plexus block.** Despite the risks of hemidiaphragmatic and upper limb paresis, a combination of a SCPB or ICPB with an ISB can provide reliable anesthesia for clavicle fracture fixation with a low incidence of conversion to GA, with multiple studies and case reports describing excellent success in surgical anesthesia provision using a SCPB in combination with an ISB. Ryan et al. (52 cases), Fugelli et al. (10 cases), Balaban et al. (10 cases), Contractor et al. (30 cases), and Reverdy et al. (12 cases) have all described 100% success in providing surgical anesthesia by combining either a SCPB or ICPB with an ISB (Table 2 and Table 3), as have a few small case series and case reports [7,24,25,32,38,39,45,46,48,51]. Three small studies reported a low block failure rate requiring conversion to GA (3 to 6%) with this combination; however, the lack of technical detail makes it difficult to provide insight if this might be associated with the level of cervical plexus blockade [8,37,43]. An RCT (60 cases) by Neha Gupta et al. (Table 2 and Table 3) compared combined cervical plexus blockade with ISB versus ISB alone as surgical anesthesia for clavicle surgery under sedation. They found that 16.6% of patients who received an ISB alone required supplementary LA at the medial end of the clavicle, while 10% required conversion to GA, unsurprising considering the absence of supraclavicular nerve blockade from a cervical plexus block [36]. In a retrospective observational study by Ryan et al., patients undergoing clavicle surgery with an ISB under GA required a very small, but statistically significantly higher dose of intraoperative fentanyl, versus those who underwent surgery under RA (SCPB and ISB) alone [25].

Patients who receive a cervical plexus block plus ISB have reduced postoperative pain scores, shorter post-anesthetic recovery times, and lower total postoperative opioid consumption than those who underwent surgery under GA alone [8].

A prospective case-control study by Olofsson et al. compared GA with ISB (50 cases) versus GA alone (76 cases) and found significantly reduced intraoperative and postoperative opioid requirements and lower pain scores in the post-anesthetic care unit, as well as a reduced incidence of postoperative nausea and vomiting [24]. However, there were no studies comparing ISB versus a cervical plexus block for patients undergoing clavicle fixation surgery under GA.

**Selective regional blocks—supraclavicular nerve, selective nerve root blocks, and other approaches to the brachial plexus.** In order to achieve reliable anesthetic and analgesic cover with minimal motor blockade, alternatives which can provide comparable cover provided by the ISB are required. One approach is to leverage on ultrasound to perform more selective, or more distal variations of the cervical plexus block or interscalene approach. These include a selective supraclavicular nerve block (versus cervical plexus block), C5 and/or C6 nerve root injections, superior trunk blocks, or supraclavicular brachial plexus blockade (Table 2 and Table S2) [9,10,26,32,43,47,50,54,59].

Diwan et al. [26] used a combined supraclavicular nerve and C5 nerve block for regional anesthesia in a small case series (20 cases). In their study, one-fifth of participants had inadequate intraoperative anesthesia, of which three participants required sedation and one required conversion to GA. This might be attributed to the complex innervation of the clavicle, which receives varying contributions from the subclavian, subscapular, axillary, and lateral pectoral nerves—all of which contain axons from both C5 and C6 nerve roots [11,64]. Nonetheless, Shanthana et al. (Table 3 and S2) successfully combined selective C5 nerve root blockade with ICPB as regional analgesia for 2 patients under GA with minimal intraoperative pain, while Kline et al. described C5 nerve root and SCPB catheters for a patient who had severe postoperative pain 11 h after the clavicle fracture surgery, which might suggest that the lack of C6 blockade may be (1) sufficient for analgesia or (2) only required in certain groups, such as fracture site and configuration or individual anatomical variation [50,54]. A high-risk patient successfully underwent clavicle fracture surgery with combined C5 and C6 nerve root block and ICPB by Salvadores et al. (Table 3 and Table S2), who reported that the patient was comfortable intraoperatively and pain-free for 12 h postoperatively [47].

A case report by Pinto et al [10]. described the use of a combined supraclavicular nerve block with a superior trunk block, formed from the C5 and C6 nerve roots, for analgesia in a patient who underwent surgery under opioid-free GA, while another combined the supraclavicular nerve block with an ISB in a high-risk patient for awake, distal clavicle fracture surgery under RA (Supplementary Table S2) [40]. The use of a superior trunk block in clavicle surgery is also described in two additional case reports apart from the aforementioned by Pinto et al. [9,10,42]. Both described its successful use as an anesthetic technique in patients undergoing clavicle or acromioclavicular joint fixation under sedation (Supplementary Table S2) [9,42].

We found two studies describing supraclavicular brachial plexus blocks with ICPB as surgical anesthesia for clavicle fracture surgery—a single case report by Baran et al. (Table 3 and Table S2) and a prospective randomized comparative study (60 cases) by Rajbanshi et al. (Table 2 and Table 3and Table S2) [32,43]. This approach targets the distal trunks or proximal divisions, which might be distal to the origin of branches, such as the subclavian nerve, compared to an ISB or superior trunk approach; however Rajbanshi et al.’s study appears to have some success with comparable GA conversion rates, and there was no statistically significant difference in complications when comparing a supraclavicular approach versus an ISB, although we note the large LA volumes used in both block groups [43].

**Fascial plane blocks.** The use of ultrasound guidance has facilitated the emergence of fascial plane blocks for various procedures. Fascial plane blocks are attractive as they avoid needle advancement towards a neural structure, and generally do not produce motor blockade [13]. Deposition of LA into fascial planes produce a blockade of local, interfascial neural structures, as well as those within adjacent muscle via bulk flow and diffusion [76]. Amongst these, CPB for clavicle fracture surgery was first described by Valdes in 2017, followed by a number of case series/reports (Supplementary Table S2) [14,15,28,29,30,77].

Two years following the original description, Roques et al. presented a combined CPB and cervical plexus block technique, which is performed by injecting LA deep into the clavipectoral fascia on both medial and lateral aspects of a midshaft clavicular fracture [78]. Rosales and Aypa, as well as Ince et al., subsequently reported its use as an anesthetic technique for clavicular surgery, without the cervical plexus component [14,20]. All reports and studies which have utilized a CPB (with or without cervical plexus blockade) in awake or sedated patients have been performed in patients with midshaft fractures, in line with the opinion of Ince et al. that CPB can only be used as anesthesia if the fracture is at the midshaft [13,14,20,78].

Atalay et al. [15,31] used a single-injection approach for the CPB (20 mL 0.25% bupivacaine). In both studies, patients remained pain-free until 12–24 h postoperatively. This single-injection approach was replicated by two other studies for patients undergoing GA, either as a sole regional technique or combined with an ISB, and all patients did not require opioids post-operatively [29,30]. Subsequent reports using CPB and GA also showed excellent intra-operative and post-operative pain control without the need for opioid rescue analgesia [28,29,30]. The report by Yoshimura and Morimoto utilized a dual injection CPB plus cervical plexus block for two cases who underwent surgery under GA. The time to first request for analgesia was 13 h to 1 day, highlighting a long duration of postoperative analgesia consistent with that reported by Atalay et al., and by Rosales and Aypa [15,20,28,31]. Most recently, Zhou et al. conducted a RCT comparing ICPB with ISB versus ICPB and CPB in awake patients undergoing midshaft clavicle fracture fixation (Table 2 and Table 3) [13]. This study is of note as the group receiving an ICPB and CPB had significantly less impairment in hemidiaphragmatic function and pulmonary function and paucity of upper limb motor blockade with no differences in block onset, pain scores in recovery, or block failure rates [13].

Another fascial plane block which has been described in clavicle fracture surgery is the PECS block. Schuitemaker et al. reported the analgesic effect of PECS II block in seven cases under GA (Supplementary Table S2) [16]. One patient was administered a PECS II block post-operatively as a rescue analgesic with improvement within 15 min. All other patients, except one, underwent GA with a modified PECS II block, and were comfortable, apart from one who required rescue morphine post-operatively, following acromioclavicular joint dislocation and hook plate fixation [16]. Subsequently, Sanllorente-Sebastian et al. also demonstrated a successful intra- and post-operative analgesia with a combination of a cervical plexus block and PECS I block for distal clavicle surgery. However, the patient reported mild post-operative pain, and she required a small dose of rescue morphine (Supplementary Table S2) [33]. However, the mechanism of the PECS block does warrant some consideration. While blockade of the medial and lateral pectoral nerves is achieved, and the latter does contribute to innervation of the clavicle and shoulder, is blockade of these nerves and bulk flow of LA alone sufficient for analgesia in clavicle surgical procedures [68]? In addition, the pectoral nerves are motor nerves, and reduction in shoulder adduction has been demonstrated previously; however, the lateral pectoral nerve, which pierces the clavipectoral fascia, should theoretically be blocked by a CPB, although motor weakness has not been demonstrated with this block [13,79].

**Local infiltration analgesia.** A trend towards performing minor limb surgeries under LA alone, such as the wide-awake local anesthesia no tourniquet (WALANT) technique has been garnering increasing interest amongst surgeons, and has been reported in clavicle surgeries. This technique utilizes a tumescent injection of lignocaine and adrenaline to provide both LA and vasoconstriction. Subcutaneous infiltration produces nociceptive blockade, and subperiosteal infiltration has been thought to facilitate LA spread via nutrient or transcortical vessels from the periosteum into the endosteal circulation, thus, blocking sensory fibers in the periosteum, mineralized bone, and bone marrow [22,27,80,81,82,83,84,85,86].

Two reports [22,27] demonstrated the potential of local infiltration as a viable anesthetic technique (Supplementary Table S2). Niempoog et al. used 50 mL 1% lignocaine with adrenaline and sodium bicarbonate for clavicle fracture fixation with no intraoperative pain and no reported complications [22]. A series of 16 cases was also reported by Ahmad et al. [27] who successfully used 40 mL 1% lignocaine with adrenaline in patients without sedation or conversion to GA. Only two patients had mild pain (2/10) during reduction. No motor block was reported, although we note the short duration of analgesia of up to 2 h post-operatively [27].

**Motor blockade and other adverse effects.** A paucity of motor blockade is desirable for the preservation of upper limb function and the prevention of respiratory compromise from phrenic or recurrent laryngeal nerve blockade, and seeking techniques which are motor-sparing is, thus, of importance to facilitate recovery whilst providing good early postoperative analgesia [17]. The interscalene approach and other approaches which target the brachial plexus or its origin nerve roots (e.g., superior trunk and selective C5 or C6 nerve root blocks) would, unsurprisingly, be associated with motor block, resulting in reduced shoulder mobility. A supraclavicular brachial plexus block might produce even more extensive motor blockade and functional impairment. No major complications were reported in the studies included in this scoping review. Amongst the included studies, few evaluated motor blockade as an outcome measure

Zhou et al. comprehensively evaluated upper limb paresis using a 3-point scale for each of the five major nerves arising from the brachial plexus, in which they found dense motor blockade persisting beyond 4 h post-block [13]. Diwan et al. reported half of the successful blocks with a combined selective C5 nerve root and supraclavicular nerve block developed shoulder and elbow weakness, defined as motor power <2/5 (Medical Research Council scale for muscle power) [26]. Using a mixture of 7.5 mL 2% lignocaine and 7.5 mL 0.5% bupivacaine for an ISB, plus another 10 mL of the same mixture in a cervical plexus block, Kaciroglu et al. found a mean duration of motor block lasting 213 min [37]. Motor block lasted almost 2 h longer (mean 347 mins) in another study by Rajbanshi et al. (Table 2 and Table 3) using a slightly larger volume of 30 mL bupivacaine 0.25% for a combined ISB and ICBP technique [43]. The same study found an even more pronounced block duration with a supraclavicular brachial plexus approach (mean 392 min) [43]. 

Approaches, such as the ISB, may also entail other undesirable adverse effects, such as phrenic nerve palsy, dysphonia, and Horner’s syndrome. Zhou et al. reported a much higher (50%) incidence of hemidiaphragmatic paresis in patients receiving and ISB combined with in ICPB [13]. The same group also described a decrease in forced expiratory volume in 1 s (FEV_1_) and peak expiratory flow rate, at 4 h post-block (reductions of 40.0% 29.3% respectively compared to pre-block) with an injectate of 20 mL [13]. Abdelghany et al. (Table 2 and Table 3) reported a statistically significant increase in phrenic nerve palsy—based on perioperative ultrasound assessment of diaphragmatic excursion, when an ISB was added to an ICPB (22.9% vs. 2.9%). However, the group administered a moderate 15 mL bupivacaine 0.25% for the ISB, and 10 mL for the ICPB [21].

The incidence of Horner’s syndrome was reported to be 5.7% with a combined SCPB and ISB technique versus 2.9% with ICPB alone by Abdelghany et al. (Table 2 and Table 3) [21]. Neha Gupta et al. reported a much higher overall (36.7%) incidence of hoarseness in their study, comparing ISB alone versus combined ISB and a cervical plexus block. Our contention is the rather large LA volumes (>20 mL) plus a lack of ultrasound guidance when performing the ISB contributed to this [36]. In their study (n = 30), Contractor et al. reported the incidence of Horner’s syndrome and hoarseness of voice to be 26.7% and 16.7%. respectively. Injectate volumes were similar to that by Neha Gupta et al. (10–15 mL 1.5% lignocaine with adrenaline plus 5–10 mL 0.5% bupivacaine), but the halved incidence of dysphonia might be due to the employment of ultrasound guidance by Contractor et al. [36,49]. In their study, Rajbanshi et al. (Table 3), reported a higher incidence of Horner’s syndrome when adding an ISB to an ICPB, as compared to ICPB with a supraclavicular brachial plexus block (7.3% versus 1.8%) [43]. The incidence of hoarseness was also higher in the group receiving the ISB (7.3% versus 3.6%) [43]. Unsurprisingly, studies reporting adverse events utilized an ISB, further compounded by (1) high LA volumes and (2) use of a nerve stimulator rather than ultrasound guidance. The incidence of such events appear to be lower with a cervical plexus block alone.

**Choice of regional technique.** A SCPB or ICPB appears to be the preferred regional block of choice for clavicle fracture surgery. However, the choice between a SCPB and an ICPB is contentious. The investing fascia that dichotomizes the SCPB and ICPB has been considered a potential barrier to deeper spread of LA compared to a subcutaneous injection. However, this fascia has also been found to be porous or lacking by histological studies, and studies in carotid and thyroid surgical patients have found no significant difference in efficacy between the two [56,57,58]. Nonetheless, considering the findings by Arjun et al., there might be an advantage of an ICPB over a SCPB, as ICPB would provide a better success rate than SCPB when combined with ISB in the context of clavicle fractures or surgery [56,57,58].

Furthermore, SCPB or ICPB is a simple regional technique which preserves upper limb motor function and avoids hemidiaphragmatic paresis, whilst providing sufficient analgesia for clavicle surgery under GA with a good safety profile, which can also be safely performed as analgesia for clavicle fracture in an emergency department setting [44,49]. Although Olofsson et al. reported benefits in adding an ISB to patients undergoing clavicle fixation under GA, a point of contention is whether the analgesic cover of an ISB, compared to a cervical plexus block, in the context of a patient under GA, is sufficiently superior to justify its inherent risks [24].

In patients in whom RA is being employed as the anesthetic technique, combined ICPB with ISB can provide reliable surgical anesthesia for clavicle fracture surgery with low incidence of block failure requiring conversion to GA. However, the ISB carries a propensity for adverse events including hemidiaphragmatic paresis, Horner’s syndrome, and hoarseness, as well as shoulder weakness. Furthermore, the potential for neural injury warrants careful consideration when including an ISB as part of the anesthetic or analgesic regimen. If further studies can replicate the experience of Zhou et al. in using a ICPB plus CPB combination, this would add a useful technique into the anesthesiologist’s armamentarium [13].

The exact site of LA deposition in a CPB warrants further discussion. The clavipectoral fascia lies below the clavicular head of the pectoralis major muscle, superficial to the subclavius, and occupies the space between the pectoralis minor and clavicle [14,20,77,78,87]. Medially this fascia fuses with the external intercostal membrane, and is bounded laterally by the coracoid process [14,87]. Several structures pierce this fascia, among which include the lateral pectoral nerve and nerve endings that innervate the clavicle [20,87]. Deposition of LA into this enclosed space is probably key to achieving adequate spread and blockade of sensory conduction, and, thus, the exact location of the needle tip is of import. Accompanying sonographic images in included studies have depicted LA deposition on either the inferior surface or anterior surface of the clavicle [13,20,28,30]. The clavipectoral fascia splits to enclose the subclavius. Descent of the subclavius can represent LA deposition either above or below the clavipectoral fascia, and might not be indicative of injectate spread into the space enclosed by the fascia, as it divides inferior to the subclavius muscle and clavicle; thus, we opine that the needle tip should be placed in close proximity to the clavicle, at the antero-inferior to anterior surface of the clavicle [13,20,28].

Local infiltration analgesia may be a viable option for surgical fixation, although the limited studies make it difficult to recommend as an anesthetic technique at this point. However, this might be a consideration for a very small group of patients where the risk of a GA is prohibitively high, and specific RA techniques for awake clavicle surgery either remain contraindicated or expertise is unavailable. Nonetheless, subcutaneous and subperiosteal infiltration of LA could be useful as an opioid-sparing technique in patients undergoing surgery under GA who are not candidates for a block.

**Moving forwards—what next?** There is currently insufficient evidence to recommend novel techniques, such as the aforementioned selective nerve blocks or fascial plane blocks. However, results from the few early studies do lend weight to some of these techniques.

The superior trunk block targets its namesake—formed by the fusion of the C5 and C6 nerve roots, with an injection site more distant from the phrenic nerve compared to the traditional ISB approach—and has been shown to produce results with significantly reduced hemi-diaphragmatic paresis compared to the ISB [64,88]. We opine, based on extrapolation from studies in shoulder surgeries, that in the context of clavicle surgery, superior trunk block can be a safe alternative to ISB, particularly when combined with an ICPB. although this has been confined to three case reports, and further studies are required [9,10,42,80]. The CPB is another promising motor-sparing regional technique alternative, based on the success from preliminary reports. Fascial plane blocks, such as CPB, additionally avoid the need to inject near a neural structure and risk neuropraxia or nerve injury, and avoid blockade of motor innervation. In combination with an ICPB, CPB holds promise in providing comparable anesthesia and analgesia to the preferred combination of a cervical plexus block with ISB [13]. Further RCTs versus GA alone, or non-inferiority examining the efficacy of blocks, such as the CPB, in isolation or compared with existing techniques, might be useful moving forwards.

In addition, there is a lack of studies examining the effect of addition of adjuncts, such a Dexamethasone or Clonidine, on the efficacy of nerve or fascial plane blocks in the context of clavicle fractures.

More observational and randomized studies are required to determine the efficacy and safety of various techniques, particularly in newer techniques, such as the CPB. Future studies should also move beyond pain scores and examine other outcomes, such as analgesic duration, return to function, patient satisfaction, and adverse events, in a structured manner. Future studies might also aim to elucidate the required concentration and safe dose of LA for local infiltration.

**Study strengths and limitations.** Our review included studies and subjects from a diverse and heterogeneous population. Furthermore, the complex sensory innervation in the clavicular region results in a permutation of different techniques used either as the main anesthetic technique or as an adjunct to general anesthesia. Furthermore, a significant proportion of included articles seem to stem from lower-tier publications, which is unsurprising given the small sample sizes. Nonetheless, we were able to collate the results in a structured manner and draw conclusions that can be used as a basis for future larger, well-designed clinical studies.

## 5. Conclusions

Current evidence of regional anesthetic and analgesic techniques in clavicle surgery is heterogeneous, congruent with different approaches used to overcome the overlapping sensory innervation. The literature largely comprises case reports and case series, with several published RCTs. Based on our scoping review, we opine that ICPB is currently the preferred regional block of choice for clavicle surgery. Multiple studies have demonstrated a combination of ICPB with ISB to be a reliable choice as surgical anesthesia. However, based on existing studies, the undesirable propensity of ISB for upper limb and phrenic nerve paresis might preclude its routine use, or make it unsuitable for certain patient groups. The clavipectoral fascial plane block is an emerging motor-sparing regional technique that might prove to be a promising motor-sparing alternative to the ISB, and further studies are eagerly awaited. More observational and randomized studies are required to determine the efficacy and safety of CPB and surgical local infiltration analgesia.

## Figures and Tables

**Table 1:** Inclusion and exclusion criteria for eligibility.

Criteria
Inclusion
Any published primary prospective or retrospective studies, case reports, case series, conference abstracts Regional anesthesia technique(s) administered for clavicular pain in the context of a clavicle fracture or surgical procedure
Exclusion
Studies on non-human subjects
Cadaveric studies
Studies not reporting clinical data or patient outcome (e.g., editorials or technical descriptions)
Overlapping participant data
Retracted articles

**Table 3:** Reclassification of cervical plexus blocks into superficial, intermediate, and deep based on a standardized nomenclature system [7,8,13,15,21,23,25,28,32,33,34,36,37,38,39,42,43,45,46,47,48,49,50,51,52,53,59,60].

Reference (Year), Country	Block Description	Needle Guidance	Original Description	Block Site (Needle Tip Position)/Description from Cited Literature
Superficial cervical plexus				
[25] Ryan and colleagues (2020), USA	In-text	LM	Superficial	Subcutaneous infiltration along the posterior border of the SCM.
[39] Kiran Kumar and colleagues (2019), Nepal	In-text	US	Superficial	Just beneath the skin, at the midpoint of the line joining the mastoid and clavicle.
[53] Kuchyn (2013), Russia	In-text	LM	Superficial	Subcutaneous infiltration along the posterior border of the SCM.
[59] Kline (2013), USA	In-text, image	US	Superficial	Scan plane along the long axis of the SCM. Hydrodissection along the superficial cervical plexus plane. Sonographic image provided, demonstrating LA deposition above the posterior border of the SCM.
Intermediate cervical plexus				
[13] Zhuo and colleagues (2022), China	In-text	US	Intermediate	Along the posterior border of SCM, into the interfascial space between the SCM and the prevertebral fascia
[21] Abdelghany and colleagues (2021), Egypt	In-text	US	Superficial	Just superficial to the prevertebral fascia.
[23] Arjun and colleagues (2020), India	In-text	US	Superficial vs. Intermediate	Study comparing SCPB vs. ICPB. SCPB consisted of subcutaneous infiltration along the posterior border of the SCM, while ICPB consisted of local anesthetic deposited after piercing the investing layer of cervical fascia.
[32] Baran and colleagues (2020), Turkey	In-text	US	Superficial	Needle inserted lateral to medial through the thyroid cartilage with the needle tip tracked under and positioned in the fascia deep to the SCM.
[38] Fugelli and colleagues (2019), Norway	In-text	US	Superficial	Local anesthetic deposited under the posterolateral belly of the SCM; sonographic image provided.
[42] Paul and colleagues (2019), India	In-text	US	Superficial	Infiltration at the posterior border of the SCM but superficial to the prevertebral fascia.
[43] Rajbanshi and colleagues (2018), Nepal	In-text	US	Superficial	Injection along the fascial plane separating the posterior border of the SCM and anterior scalene muscle.
[44] Ho and colleagues (2018), Canada	In-text, image	US	Superficial	The needle is visualized in position just deep to the lateral border of the SCM with injectate seen tracking along the fascial plane.
[45] Balaban and colleagues (2018), Turkey	In-text	US	Not specified	Plane block in the prevertebral fascia posterior to the SCM. Needle advanced along the posterior border of the SCM to the nerve point of the neck.
[46] Shrestha and colleagues (2017), Nepal	In-text, image	US	Superficial and intermediate	Both SCPB and ICPB was performed. Injection performed just beneath the skin at the lateral border of the SCM. Additionally, for the second case, injectate was deposited at the superficial cervical plexus (indicated on the provided image to be superficial to the prevertebral fascia and anterior and middle scalene muscles, and deep to the SCM).
[47] Salvadores de Arzuaga and colleagues (2017), Spain	In-text	US	Superficial	Beneath the posterior border of the SCM, above the prevertebral fascia, and avoiding excessive medial spread of the injectate.
[7] Reverdy (2015), France	In-text, image	US	Superficial	Injection between the anterior and middle scalene muscles, in the space posterior to the SCM.
[49] Flores and colleagues (2015), USA	In-text, image	US	Superficial	Injectate deposited under the SCM, in the fascial space between the SCM and levator scapulae muscles.
[50] Shanthanna (2014), Canada	In-text	US	Superficial	Needle positioned just under the SCM, at the posterior border around the midpoint between C6 and the mastoid process.
[52] Dillane and colleagues (2014), Canada	In-text	US	Superficial	Injectate deposited deep to the prevertebral fascia between the SCM and anterior scalene muscles *.
Deep cervical plexus				
[8] Banerjee and colleagues (2019), India	In-text	US	Superficial	Needle position under the SCM below the prevertebral fascia.
[51] Vandepitte and colleagues * (2014), USA	In-text	US	Superficial	Needle beneath the prevertebral fascia.
Technique not described or in insufficient detail				
[28] Yoshimura and Morimoto (2020), Japan	NR	NR	Superficial	Not reported or directly referenced.
[31] Atalay and colleagues (2020), Turkey	NR	US	Superficial	Not reported or directly referenced.
[33] Sanllorente-Sebastián and colleagues (2020), Spain	NR	LM	Superficial	Not reported or directly referenced.
[34] Ho and colleagues (2020), Canada	NR	US	Superficial	Not reported or directly referenced.
[36] Neha Gupta and colleagues (2019), India	NR	NR	Superficial	Not reported or directly referenced.
[37] Kaciroglu and colleagues (2019), Turkey	NR	US	Superficial	Not reported or directly referenced.
[48] Contractor and colleagues (2016), India	NR	Unclear, possibly US	Superficial	Not reported or directly referenced.
[60] Choi and colleagues (2005), USA	NR	NR	Deep and Superficial	Not reported. Superficial component described as a classic approach with reference to a single article †.

Abbreviations are as follows: US, ultrasound; SCM, sternocleidomastoid; LM, landmark; LA, local anesthetic; SCPB, superficial cervical plexus block; ICPB, intermediate cervical plexus block; NR, not reported. * A series of 10 cases of clavicular fixation performed under interscalene block as the sole anesthetic modality was briefly mentioned in this case report by Vandepitte and colleagues with no further details provided. † Not described in detail in-text. The authors report it as a classic approach, with a single reference to: Adriani J. Blocking of spinal nerves. In: Adriani J, ed. Labat’s *Regional anesthesia: techniques and clinical applications*. St. Louis: Warren H. Green, 1985:236–54 [60]. This book chapter provides a few different approaches to the cervical plexus, and there is insufficient information to identify the exact technique used.

## Data Availability

The data presented in this study are available on request from the corresponding author.

## Supplementary Materials

The following supporting information can be downloaded at: https://www.mdpi.com/article/10.3390/healthcare10081487/s1, Table S1: Search terms employed in the electronic database search strategy; Table S2: A summary of case report/case series included in this scoping review. References [9,10,14,15,16,20,22,26,27,28,29,30,32,33,34,38,39,40,41,42,46,47,49,50,51,52,59,60] are cited in supplementary.
