# Supplementary material for: Regional Anesthetic and Analgesic Techniques for Clavicle Fractures and Clavicle Surgeries: Part 1—A Scoping Review
Source: Healthcare (Basel). 2022 Aug 7;10(8):1487. doi: 10.3390/healthcare10081487 (PMC9408139; doi:10.3390/healthcare10081487)
Supplement: Supplementary file 1 [file healthcare-10-01487-s001.zip › healthcare-1729411 updated supplementary 1.pdf]

Table S1. Search terms employed in the electronic database search strategy.

No.	Search terms
	Clavicle fracture
1	Exp Clavicle/
2	Fracture*.tw
3	1 AND 2
4	Clavic* fracture*.tw
5	1 OR 3 OR 4
	Regional anesthesia
6	Exp Nerve Block/
7	Regional anesth*.tw
8	Anesth*.tw
9	Exp Acute Pain/
10	Exp Pain, Postoperative/
11	Exp Analgesia/
12	6 OR 7 OR 8 OR 9 OR 10 OR 11
	Combined search concept
13	5 AND 12
