# Supplementary material for: Regional Anesthetic and Analgesic Techniques for Clavicle Fractures and Clavicle Surgeries: Part 1—A Scoping Review
Source: Healthcare (Basel). 2022 Aug 7;10(8):1487. doi: 10.3390/healthcare10081487 (PMC9408139; doi:10.3390/healthcare10081487)
Supplement: Supplementary file 1 [file healthcare-10-01487-s001.zip › healthcare-1729411 updated supplementary 2-forMerrin.pdf]

Table S2. A summary of case report/case series included in this scoping review. References [9,10,14–16,20,22,26–30,32–34,38–42,46,47,49–52,59,60] are cited in supplementary. Abbreviations: *ICPB, Intermediate Cervical Plexus Block; ISB, Interscalene Brachial Plexus Block; CPB, Clavipectoral Plane Block; COX, Cyclooxygenase, RCT, Randomized Controlled Trial; LA, Local Anesthetic; NR, Not Reported; SCPB, Superficial Cervical Plexus Block; SpC, Supraclavicular Brachial Plexus Block; WALANT, Wide-Awake Local Anesthesia no Tourniquet; DCP, Deep Cervical Plexus; SpN, Supraclavicular Nerve; GA, General Anesthesia; NB, Nerve Block; ACJ, Acromioclavicular Joint; PECS, Pectoralis Nerve; VAS, Visual Analogue Scale; NRS, Numerical Rating Scale; POD, Postoperative Day; PCA, patient controlled analgesia; IV, Intravenous.*

Reference (year), country	Study design	Sample size	Fracture Location	Type of Block	Needle guidance	LA type and volume	Anesthetic Technique	Multimodal analgesia regime	Outcome(s)
[9]	Case report	1	Distal	Superior trunk block	US	8mL Ropivacaine 0.75%	Sedation	Paracetamol, ketorolac 30mg	Comfortable intraoperatively.
[10]	Case report	1	Proximal	SpN and Superior trunk block	US	SpN: 2mL Ropivacaine 0.75%<br>Superior trunk block: 10mL Ropivacaine 0.75%	GA	NR	1. No intra-operative opioids.<br>2. No pain in recovery.
[14]	Case report	1	Midshaft	CPB	US	30mL Ropivacaine 0.25% and Lignocaine 1%	Awake	Paracetamol, PCA fentanyl	1. Pre-operative NRS 8/10 pre-block to 0/10 post-block.<br>2. Pain scores NRS ≤4/10 up to 24 hours post-operatively.<br>No reported complications.
[15]	Case report	1	NR	SCPB and CPB	US	SCPB: NR<br>CPB: 20mL 0.25% Bupivacaine	GA	Ibuprofen	No pain (NRS 0/10) in the post-anesthetic care unit.<br>No pain until 24 hours after surgery.<br>No reported complications.
[16]	Case series	7	NR, 4 ACJ dislocations	PECS II	US	0.9 to 1.3mg.kg^{-1} Bupivacaine 0.375% to 0.5% with Adrenaline or 1.3mg.kg^{-1} Levobupivacaine	GA	NR	1. All comfortable postoperatively except one case (ACJ dislocation and hook plate) which required rescue Morphine in recovery.

[20]	Case Report	1	Midshaft	CPB	US	0.5% with Adrenaline 15mL 0.25% Levobupivacaine and 15mL 1% Lignocaine	Sedation	Paracetamol , COX-2 inhibitor	1. Surgery under sedation. 2. First onset of pain at 16 hours post-block.
[22]	Case report	1	Midshaft	WALAN T	LM	50mL Lignocaine 1% with Adrenaline and sodium bicarbonate	Awake	Naproxen, paracetamol	No pain intra-operatively
[26]	Case series	20	14 Midshaft 6 Distal	SpN and C5 NB	US	SpN: 3mL Bupivacaine 0.5% C5 NB: 3mL Bupivacaine 0.5%	Awake	Fentanyl	1. Inadequate anesthesia in 4 (20%) patients. 2. One conversion to GA, 3 patients completed surgery with sedation. 2. Shoulder weakness in 50% of successful blocks. 3. Sensory block duration 5.5 to 12 hours.
[27]	Case series	16	NS	WALAN T	LM	40mL Lidocaine 1% with Adrenaline 10 mL subcutaneously along the incision site followed by 30mL subperiosteally at multiple intervals and directions (2mL subperiosteally and 4mL anteriorly, 4 mL posteriorly)	Awake	NR	1. No pain during surgery except in 2 patients (NRS of 1 to 2) during reduction 2. No motor block. 3. Analgesia up to 2 hours after surgery
[28]	Case series	2	NR	SCPB and CPB	NR / US	SCPB: NR CBP: 15mL Levobupivacaine 0.375%	GA	NR	1. Minimal post-operative pain (NRS 0-2/10). 2. No analgesia used until 13h or next day.

[29]	Case series	4	Midshaft	CPB	US	10-15mL Levobupivacaine 0.4%	GA	Paracetamol, parecoxib	Good pain control without intraoperative or postoperative opioid rescue.
[30]	Case series	3	1 Medial 1 Midshaft 1 Distal§	CPB§	US	CPB: 10-15mL Ropivacaine 0.5%	GA	NR	No post-operative surgical site pain¶.
[32]	Case report	1	NR	SCPB and SpC	US	SCPB: 10mL Bupivacaine 0.5% with 5 mL Lidocaine 2% SpC: 5 mL Bupivacaine 0.5% with 5 mL Lidocaine 2%	Sedation	NR	No intra-operative pain.
[33]	Case report	1	Distal	SCPB and PECS I	LM / US	SCPB: 5mL Ropivacaine 0.5% PECS I: 15mL Ropivacaine 0.5%	GA	Paracetamol and dexketoprofen	Mild post-operative pain (NRS 2-3/10) in recovery.
[34]	Case report	1	NR	SCPB and ISB	US	SCPB: 10mL Ropivacaine 0.5% ISB: 30mL Ropivacaine 0.5%	GA	Paracetamol, hydromorphone	1. No immediate post-operative pain in recovery 2. Hoarseness without dyspnea or Horner's syndrome. 3. Discomfort at 15 hours post-operatively.
[38]	Case series	10	Midshaft	SCPB and ISB	US	Ropivacaine 0.75%, mean volume 30.8mL	Sedation	NR	No intra-operative opioids or rescue LA.
[39]	Case series	3	NR	SCPB and ISB	US	SCPB: 15mL Bupivacaine 0.25%	Sedation	NR	Comfortable intraoperatively.

						with 2mg Dexamethasone ISB: 25mL Bupivacaine 0.25% with 4mg Dexamethasone			
[40]	Case report	1	Distal	SpN and ISB	US	SpN: 4mL Bupivacaine 0.5% ISB: 10mL Bupivacaine 0.5%	Awake	NR	1. No intra-operative sedatives or analgesics. 2. No analgesics in the first 12 hours.
[41]	Case report	1	NR	SpN and ISB	US, PNS	SpN: 5mL Ropivacaine 0.375% ISB: 30mL Ropivacaine 0.375%	Sedation	NR	1. No intra-operative pain. 2. Ipsilateral vocal cord paralysis, hoarseness, and dysphagia lasting 8-12 weeks. 3. During a repeat surgery for implant removal, the procedure was repeated with much smaller volumes with no complications. (4mL for SpN block and 3mL for superior trunk block)
[42]	Case report	1	ACJ	SCPB and Superior trunk block	US	SCPB: 5mL Levoupivacaine 0.375% Superior trunk block: 12mL Levobupivacaine 0.375%	Sedation and subarachnoid block	NSAIDS	1. Polytrauma patient undergoing concurrent right ACJ repair and right anterior cruciate ligament repair. 2. Comfortable intraoperatively, pain-free for 8 hours (site not specified).
[46]	Case series	2	NR	SCPB and ISB	US, PNS	SCPB: 15 to 20mL Ropivacaine 0.25% with 15-30μg Dexmedetomidine	Sedation	Ketorolac	Analgesia duration 6 to 10 hours

						ISB: 24 to 30mL Ropivacaine 0.25% with 30-40μg Dexmedetomidine			
[47]	Case report	1	Midshaft	SCPB and C5, C6 NB	US	SCPB: 5mL Bupivacaine 0.5% C5, C6 NB: 2mL Bupivacaine 0.5%	Sedation	Fentanyl	1. Comfortable intraoperatively. 2. Pain-free for 12 hours.
[49]	Case report	1	NR	SCPB	US	SCPB: 10mL Bupivacaine 0.5%	NA	NR	1. Emergency department report, block performed for analgesia. 2. Complete pain relief in 15 minutes, and development of Horner's syndrome in 45 minutes, which resolved in 90 minutes.
[50]	Case series	2	NR	Selective C5 NB and SCPB	US	C5 nerve block: 2mL Ropivacaine 0.5% SCPB: 8mL Ropivacaine 0.5%	GA	Fentanyl	1. Minimal intra-operative pain, and NRS <2 in recovery.
[51]	Case report	1*	Midshaft	SCPB and ISB†	US, PNS	SCPB: 5mL Ropivacaine 0.5% ISB: 15mL Ropivacaine 0.5%	Sedation	Paracetamol, tramadol	1. Pre-operative NRS 7/10 to 0/10 post-block. 2. Uneventful surgery.
		10	NR	ISB	NR	NR	Sedation or awake	NR	NR‡
[52]	Case report	1	NR	SCPB and ISB	US	SCPB: 15mL Ropivacaine 0.5% and Bupivacaine 0.125% ISB: 5mL Ropivacaine 0.5% and Bupivacaine 0.125%	Awake	NR	Comfortable intraoperatively. Complete motor block of right upper limb.

[59]	Case report	1	Distal	SCPB catheter and C5 NB, then C5 catheter on POD 1	US	SCPB catheter: 5mL Lignocaine 2% with Adrenaline and 10mL Ropivacaine 0.5%; then 0.2% Ropivacaine 8mL/hour C5 catheter: 2% Lignocaine 3mL with Adrenaline and 8mL 2% Lignocaine; then 4mL/hour** NRS 0/10, intact distal arm motor function on infusion	GA	Fentanyl, paracetamol	1. Postoperative NRS 3/10 in recovery, then 8-10/10 at 11 hours. No motor block. 2. C5 NB nerve block catheter inserted on POD 1, post-block NRS 0/10. Distal arm motor function spared.
[60]	Case report	1	NR	SCPB and DCP	NR	Bupivacaine 0.5%. Volumes not reported.	GA	NR	Pain-free for 14 hours.

* Pregnant patient at 15 weeks' gestation.

† Interscalene technique described in the manuscript as local anesthetic deposition between superior and middle trunks.

‡ A series of 10 cases of clavicular fixation performed under interscalene block as the sole anesthetic modality was briefly mentioned in this case report by Vandepitte and colleagues with no further details provided.

§ One patient with a distal clavicle fracture had an interscalene catheter inserted in addition to the clavipectoral fascial plane block.

¶ One patient with other accident-related injuries and a pain score of 6/10 required 49.5mg of oral Morphine equivalent in the post-anesthetic care unit.

** Local anesthetic agent and concentration not specified.
